# USP15 promotes the progression of papillary thyroid cancer by regulating HMGB1 stability through its deubiquitination

**DOI:** 10.7150/jca.92386

**Published:** 2024-03-11

**Authors:** Si-si Wang, Dao-xiong Ye, Bo Wang, Meng-yao Li, Wen-xin Zhao

**Affiliations:** 1 Fujian Medical University, Fuzhou, Fujian 350001, P.R. China.; 2 Department of Thyroid Surgery, Fujian Medical University Union Hospital, 29 Xinquan Road, Fuzhou, Fujian 350001, P.R. China.

**Keywords:** PTC, USP15, HMGB1, deubiquitination

## Abstract

**Purpose:** Papillary thyroid cancer (PTC) stands as one of the most prevalent types of thyroid cancers, characterized by a propensity for in-situ recurrence and distant metastasis. The high mobility group protein (HMGB1), a conserved nuclear protein, plays a pivotal role in carcinogenesis by stimulating tumor cell growth and migration. Nevertheless, the underlying mechanism driving aberrant HMGB1 expression in PTC necessitates further elucidation.

**Materials and methods:** Our study unraveled the impact of low and overexpression of USP15 on the proliferation, invasion, and metastasis of PTC cells. Through a comprehensive array of molecular techniques, we uncovered the intricate relationship between HMGB1 and USP15 in the progression of PTC.

**Results:** In this study, we identified USP15, a deubiquitinase in the ubiquitin-specific proteases family, as a true deubiquitylase of HMGB1 in PTC. USP15 was shown to interact with HMGB1 in a deubiquitination activity-dependent manner, deubiquitinating and stabilizing HMGB1. USP15 depletion significantly decreased PTC cell proliferation, migration, and invasion. In addition, the effects induced by USP15 depletion could be rescued by further HMGB1 overexpression. But when HMGB1 is knocked down, even overexpression of USP15 could not promote the progression of PTC cells.

**Conclusion:** In essence, our discoveries shed light on the previously uncharted catalytic role of USP15 as a deubiquitinating enzyme targeting HMGB1, offering a promising avenue for potential therapeutic interventions in the management of PTC.

## Introduction

Thyroid cancer (TC) is the most common malignancy of the endocrine system, accounting for about 2.1% of all cancers worldwide 1. The incidence of TC has increased significantly worldwide in recent years and is becoming the most rapidly growing tumor 2. According to data released by the American Cancer Society in 2020, there are 52890 new cases of TC in the United States in 2020, ranking fifth among women and accounting for 4% of all cancers 3. Depending on the pathological type, TC is divided into papillary thyroid carcinoma (PTC), follicular thyroid carcinoma (FTC), medullary thyroid carcinoma (MTC), and anaplastic thyroid carcinoma (ATC), among which PTC is the most common type of thyroid cancer and comprises of more than 80% of all TC 4. While PTC patients typically face well-differentiated thyroid cancer with a relatively favorable prognosis, globally, approximately 41,000 deaths occur annually within one year due to the high incidence, recurrence, and metastasis of PTC 5. Around 20% of early-stage PTC patients experience recurrence and metastasis post-surgery. More critically, some recurrent PTC cases may transform into poorly differentiated thyroid cancer with a dismal prognosis 6. Despite the success of surgical resection, radioiodine therapy, and postoperative TSH suppression in achieving positive therapeutic outcomes in most PTC patients, a subset remains highly malignant and prone to in-situ recurrence and distant metastasis 7, 8. During treatment or natural disease progression, a subset of PTC patients undergoes regressive changes in tumor cell morphology and function, losing differentiation phenotypes. Consequently, they evolve into iodine-refractory thyroid cancer, leading to recurrence, distant metastasis, and ultimately, death 9. The invasion and metastasis of PTC is a complex multi-stage process regulated by multiple genes, multiple factors, and multiple signaling pathways, involving the decline of adhesion between cancer cells and the cell matrix, extracellular matrix degradation, tumor cell migration, distant colonization and other links 10, 11. Therefore, the molecular mechanism of invasion and metastasis of PTC has been unclear. Some studies have shown that PTC is usually characterized by inflammatory immune cell infiltration 12, relating to molecular mechanisms such as RAS gene mutation, BRAF gene mutation, RET/PTC rearrangement, PAX8-PPARγ rearrangement, and TERT promoter mutation 13. However, the potential therapeutic targets and signaling pathways are still being explored.

High-mobility group box 1 (HMGB1) is a nuclear protein that is highly conserved. It functions as a chromosome-binding protein, interacting with DNA, and plays a role in promoting the transcription of related target genes post-protein synthesis 14. HMGB1 can be secreted extracellularly and actively engages in inflammation via the RAS and MAPK pathways. This process involves the phosphorylation of MAPKs and activation of nuclear transcription factors, ultimately leading to the induction of inflammation and facilitating the migration of immune cells 15. In addition, HMGB1 plays an important role in chromatin changes, chromatin remodeling, regulation of gene transcription, and DNA replication, which can induce the growth and migration of cancer cells and participate in the progression of cancer 16, 17. In cancer cells, HMGB1 acts mainly through two signaling pathways. One pathway is that HMGB1 binds to the cell membrane surface receptor RAGE to activate its pathway and up-regulate the expression of VEGF, thus promoting tumor proliferation 18. Another pathway is that HMGB1 binds to toll-like receptors, activating the MAPK signaling pathway to induce angiogenesis, promoting tumor cell proliferation, invasion, and metastasis 19. Studies have found that HMGB1 expression is significantly up-regulated in Thyroid cancer tissues, and HMGB1 knockout significantly inhibits autophagy, sodium/iodine transporter degradation, and iodine uptake in Thyroid cancer cells treated by starvation induction 20. Moreover, It was shown that HMGB1 increases the expression of miR-221 and miR-222 in primary cultures of excised papillary lesions and in an established papillary cancer cell line (BC PAP) 21. Meanwhile, the overexpression of oncogenic miR-221 and miR-222 induced by HMGB1 is linked to heightened malignancy scores, specifically increased cell growth and motility. Additionally, the interaction of extracellular HMGB1 with RAGE enhances the expression of the oncogenic miR-221/222 cluster, subsequently suppressing the tumor suppressor gene PTEN in cell lines derived from human thyroid anaplastic and papillary cancers 22. The proposed novel pathway involving HMGB1/RAGE/miR-221/222 may provide an effective mechanism for tumors to evade immune surveillance, suggesting potential avenues for new therapeutic strategies against anaplastic tumors.

Ubiquitin is a process in which ubiquitin molecules specifically modify target proteins, and it plays a very important role in protein localization, metabolism, function, regulation, and degradation 23. Ubiquitination is closely related to the occurrence and development of malignant tumors 23. Deubiquitination is the reverse process of ubiquitination, which can play the opposite role of ubiquitination by removing the ubiquitination modification on the substrate protein 24. Deubiquitinases (DUBs) are a key factor in the regulation of ubiquitination and can be categorized into six families in the human genome: ubiquitin COOH-terminal hydrolases (UCH), ubiquitin-specific proteases (USP), the JAB1/MPN/MOV34 family (JAMM), Josephins, ovarian tumor proteases (OTU), and motif interacting with ubiquitin-containing novel DUB family (MINDY) 25. USP15 is an important member of the DUBs family 26 and is overexpressed in breast cancer, colorectal cancer, ovarian cancer, and other tumors, and is involved in the genesis and development mechanism of tumors 27-30. Recently, a study indicated that MFSD4A-AS1 activated transforming growth factor (TGF)-β signaling by sponging miR-30c-2-3p that targeted TGFBR2 and USP15, which promoted lymphangiogenesis and lymphatic metastasis of PTC 31. However, the specific role of USP15 in PTC and its relationship with HMGB1 still need to be further studied.

In the current study, the high level of HMGB1 protein expression in PTC cells was first confirmed. Subsequently, we identified the proteins interacting with HMGB1 by immunoprecipitation and mass spectrometry. Among these, USP15 was observed to be the most potent DUB responsible for HMGB1 deubiquitination and stabilization in PTC. Furthermore, we found that USP15 promotes cell proliferation, migration, and invasion through HMGB1. In summary, our findings confirmed the catalytic role of USP15 as an HMGB1 deubiquitination enzyme and provided a possible target for the treatment of PTC.

## Method and Materials

### Cell lines and cell culture

In this study, we have selected three cell lines, including, HEK-293T, B-CPAP cells, and KTC-1 cells, based on the previous study 32-34. The human embryonic kidney HEK-293T cell line was obtained from the Chinese Typical Culture Preservation Center Cell Bank. PTC cell lines, including B-CPAP cells and KTC-1 cells, were purchased from the Center of Excellence in Molecular and Cellular Science, Chinese Academy of Sciences. All cell lines were authenticated by the cell banks with short tandem repeat analysis. HEK-293T cells and PTC cells respectively were cultured in Dulbecco's Modified Eagle's Medium (DMEM, 4.5 g/L, Gibco) and Roswell Park Memorial Institute-1640 medium (RPMI-1640, Gibco), which supplemented with 10% fetal bovine serum (FBS, Gibco), penicillin G (100 U/mL) and streptomycin (100 mg/mL), under 37 °C and 5% CO_2_ culture conditions.

### Plasmid and transfection

The full-length constructs of USP15 and HMGB1 were cloned into the pcDNA3.1(+) or GV350 plasmid vector (GenChem). Knockdown of endogenous USP15 and HMGB1 was performed by cloning two short hairpin RNA (shRNA) oligonucleotides (USP15 Sh#1 and USP15 Sh#2) and one shRNA oligonucleotide (HMGB1 Sh#1) into the GV493 plasmid vector (GenChem). The sequences of the separate shRNA fragments are listed in Table S2. The deletion mutant constructs of USP15 (USP15 C298A) and His-K48 Ub (His-K48R Ub) were acquired from Addgene. HA-USP15, Flag-HMGB1, His-Ub, His-K48 Ub, and negative control were purchased from GenePharma. Transient transfections were carried out using Lipofectamine 2000 (Invitrogen) and Entranster-R4000 (Engreen Biosystem) following the protocol from the manufacturer. After transfection, the cells were treated with cycloheximide (CHX) or MG 132, respectively.

### Quantitative real-time PCR (qRT-PCR)

For qRT-PCR, the total RNA of the cells under various treatments was isolated and collected by using the TRIzol reagent (Invitrogen). The cDNA was transcribed by purified RNA utilizing a PrimerScript RT Reagent kit (TaKaRa). The qRT-PCR was performed to quantify the USP15 and HMGB1 transcript levels by using the specific primers (Table S3). The β-tubulin expression was regarded as endogenous control to calculate the target gene relative mRNA expression level. The qRT-PCR was performed as previously described 35. The experiments were repeated in triplicate to confirm the findings.

### Co-immunoprecipitation (Co-IP) and western blot

Cells were lysed with Co-IP RIPA buffer (Beyotim) containing a cocktail of protease inhibitors on ice for 30 min and centrifuged for 20 min at 12000 rpm at 4 ℃. The supernatants, the total lysed samples, were collected and precleared with IgG antibodies for 2 h. Subsequently, the samples were immunoprecipitated with indicated antibody and Protein A/G PLUS-Agarose beads (Santa Cruz) at 4 °C overnight. After washing the immunocomplexes three times with lysis buffer, the immunoprecipitated proteins were collected for western blot analysis.

### Western blot and antibodies

Whole-cell lysates (WCL) were collected using the protein lysis buffer containing proteinase and phosphatase inhibitors. The BCA assay was operated to measure the protein concentration. The protein was separated by 10% sodium dodecyl sulfate-polyacrylamide gel electrophoresis (SDS-PAGE) gels and transferred to polyvinylidene difluoride (PVDF) membranes (0.25 μm, Millipore). The membranes were then blotted with primary antibodies followed by the secondary antibody and developed with enhanced chemiluminescence reagent (Invitrogen). The primary antibodies used in this study included USP15 (67557-1-Ig, Proteintech), HMGB1 (SAB1403925, Sigma), Ub (sc-8017, Santa Cruz), HA-tag (51064-2-AP, Proteintech), Flag-tag (66008-3-Ig, Proteintech), His-tag (12698, CST), and β-tubulin (MA5-16308, Proteintech). The relative protein quantification was analyzed using ImageJ software.

### Immunofluorescence

Cells were cultured in twelve-well plates with coverage of glass cover-slips up to 40% confuency. The cells were washed and fixed with 4% paraformaldehyde for 10 min at room temperature. All slides were washed with phosphate buffer saline (PBS) and blocked with 5% bovine serum albumin (BSA) containing 0.1% Triton-100 at room temperature for 30 min. The cells were incubated with USP15 and HMGB1 primary antibodies at 4 °C overnight, washed with PBS, and then incubated with Alexa Fluor 488-conjugated anti-mouse IgG (A-21202, Thermo Fisher) or Alexa Fluor 594-conjugated anti-rabbit IgG (A-11012, Thermo Fisher) for 1 h at room temperature. After intermediate washes, fluorescent signals were detected under a fluorescence microscope (Nikon).

### Deubiquitination assay

HEK-293T cells and B-CPAP cells were co-transfected with HA-USP15, HA-USP15 C298A, Flag-HMGB1, His-Ub, His-K48 Ub, or HisK48R Ub plasmid as indicated for 48 h. His-ubiquitinated HMGB1 was immunoprecipitated with an anti-Flag antibody followed by western blot analysis. B-CPAP cells and KTC-1 cells were transfected with USP15 Sh#1 and USP15 Sh#2 as indicated for 72 h. Ubiquitinated HMGB1 was immunoprecipitated with anti-HMGB1 antibody followed by western blot analysis.

### Cell proliferation

Cell proliferation was performed by the Cell Counting Kit-8 (CCK8, Beyotime) method. Briefly, cells were seeded at a density of 1000 cells/well into 96-well plates and cultured for 24 h, 10 μL CCK8 was added to each well and incubated for 3 h. The absorbance at 450 nm was measured using a multimode reader (Molecular Devices).

### Wound healing, migration, and invasion assay

For the wound-healing assay, 1×10^6^ cells were seeded into six-well plates. When the cells reached 90% confluence, the cell layer was scratched with a 10 μL sterile plastic tip and cultured for 72 h in a medium containing 1% FBS. Images were taken under a microscope at the indicated time point to evaluate the healing rate of gap closure.

For the transwell assay, 5×10^5^ cells were seeded into the upper matrigel of a transwell chamber with 8 μm pore in a medium without FBS. The bottom chambers were filled with 600 μL fresh medium containing 10% FBS. After incubation for 48 h, the cells on the bottom membrane were fixed with 4% paraformaldehyde and stained with crystal violet. Matrigel-invading or migratory cells were counted under a microscope.

### Mass spectrometry

The LC-ESL-LTQ-Orbitrap-MS method was used to identify HMGB1-interacting proteins as described in a previous study 36. Briefly, the protein bands were cut from the gel and transferred to 100 mM NH_4_HCO_3_ with 50% acetonitrile for excising and destaining. Subsequently, the proteins were reduced, alkylated, and dried in a vacuum centrifuge. The gel pieces harboring proteins were incubated in a digestion solution at 37 °C for 24 h. The tryptic peptide mixture was purified with a ZipTipC18 microcolumn (ZTC18S096, Millipore) and subjected to separation on a Pep Map C18 trap column (75 μm, 15 cm) with column flow rates of 200 nL/min.

### Statistical analysis

Statistical analyses were performed using SPSS version 18.0 software. Data are expressed as means ± SD or means ± SEM. The student's *t*-test with one-tailed was used to compare the difference between the two groups, and the one-way ANOVA with a BrownForsythe test was accepted for multiple comparisons. Differences were considered statistically significant at a P value<0.05. Experimental data were obtained from three independent experiments unless otherwise presented. All *P<0.05, **P<0.01, ***P<0.001, ****P<0.0001, and ns: no significance.

## Results

### HMGB1 promotes the progression of PTC

**Figure 1:**
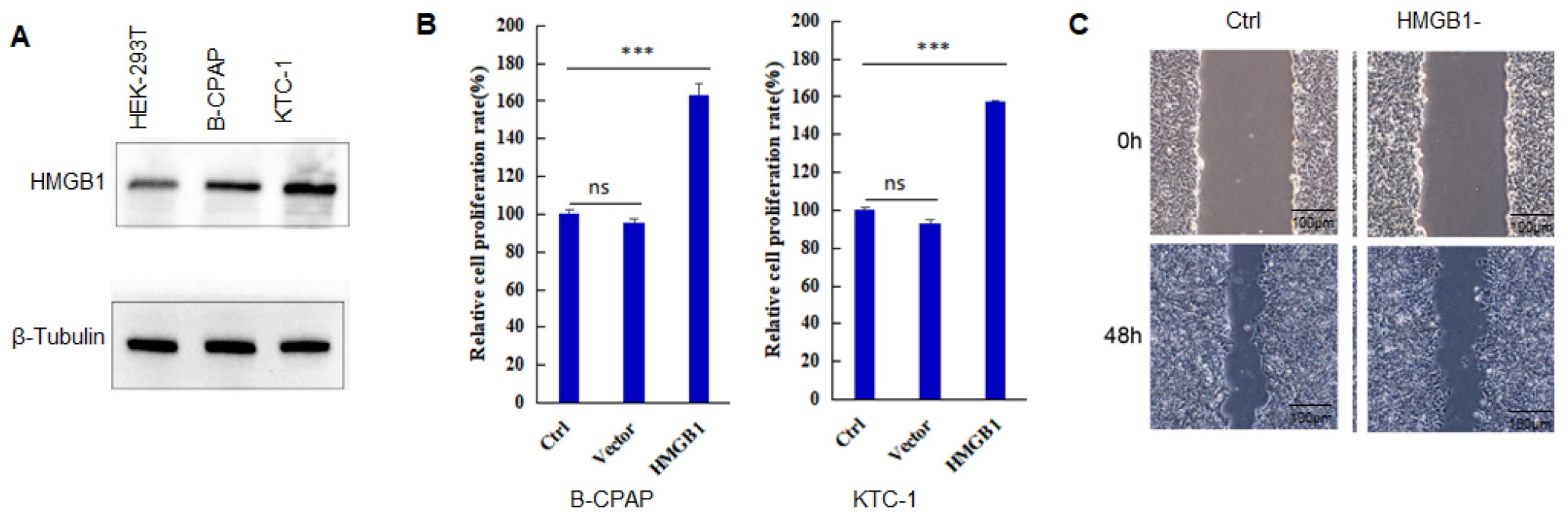
HMGB1 promotes the progression of PTC. **A:** The expression level of HMGB1 in HEK-293T cells, B-CPAP cells, and KTC-1 cells. **B:** Cell proliferation assay of B-CPAP cells and KTC-1 cells with HMGB1 overexpression. **C:** Wound-healing assay of B-CPAP cells with HMGB1 depletion. ***P<0.001 and ns: no significance.

HMGB1 expression is found in many tumors, and its level is much higher than that of corresponding normal tissues 18,19. As shown in Figure 1A, the expression level of HMGB1 protein in B-CPAP cells and KTC-1 cells is significantly higher than that in HEK-293T cells. When PTC cells overexpressed HMGB1, the proliferation capacity of the cells (B-CPAP and KTC-1) also increased significantly compared to Ctrl and Vector groups (Figure 1B) (P<0.001). After HMGB1 knockdown, the invasion capacity of PTC cells was reduced (Figure 1C). These results suggested that HMGB1 played a key role in the development of PTC.

### USP15 interacts with HMGB1

**Figure 2:**
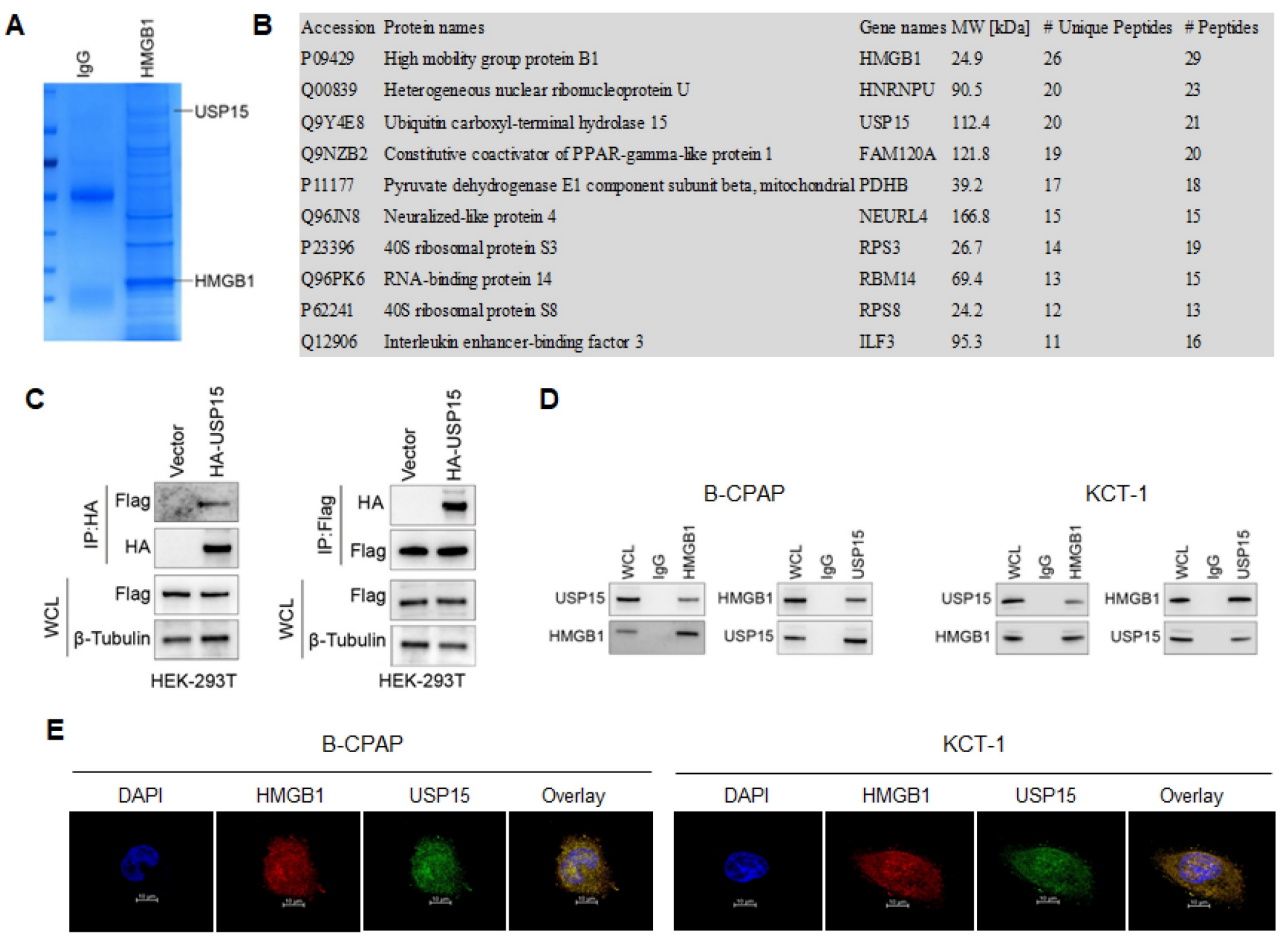
USP15 interacts with HMGB1. **A:** Coomassie brilliant blue stained protein co-precipitated with HMGB1. **B:** Mass spectrometry analysis of HMGB1-binding protein. In this study, we have listed ten potential HMGB1-interacting proteins based on the Protein peptide abundance. HMGB1, HNRNPU, and USP15 are the most enriched protein in this study. **C:** Co-IP (Co-immunoprecipitation) assay of USP 15 and HMGB1 in HEK-293T cells transfected with USP15 and HMGB1 overexpression. **D:** Co-IP assay of USP 15 and HMGB1 in B-CPAP cells and KTC-1 cells. **E:** USP15 and HMGB1 expression and subcellular localization were detected using immunofluorescence in B-CPAP cells and KTC-1 cells.

To systematically study the mechanism of HMGB1 promoting the development of TC, the interacting proteins were first identified. It was identified 302 proteins interacting with HMGB1 by Co-IP (Figure 2A). Through mass spectrometry analysis, Figure 2B lists the 10 proteins with the most binding unique peptides to HMGB1, including HMGB1, heterogeneous nuclear ribonucleoprotein U (HNRNPU), USP15, constitutive coactivator of PPAR-gamma-like protein 1 (FAM120A), pyruvate dehydrogenase E1 component subunit beta (PDHB), neuralized-like protein 4 (NEURL4), 40S ribosomal protein S3 (RPS3), RNA-binding protein 14 (RBM14), 40S ribosomal protein S8 (RPS8) and interleukin enhancer-binding factor 3 (ILF3). Among these proteins, USP15 could regulate transcriptional activity, maintain gene stability, and participate in cell cycle processes 37. Therefore, the interaction between USP15 and HMGB1 was investigated in depth (Table S1).

In Figure 2C, it is further demonstrated that USP15 can bind to HMGB1 in HEK-293T cells overexpressed with USP15 and HMGB1. Co-IP results of the endogenous proteins from B-CPAP cells and KTC-1 cells showed that USP15 could interact with HMGB1, supporting the functional cooperation of USP15 and HMGB1 (Figure 2D). In addition, results of immunofluorescence indicated that USP15 and HMGB1 at least partially co-localized in B-CPAP cells and KTC-1 cells (Figure 2E).

### USP15 modulates HMGB1 stability in a DUB-dependent manner

**Figure 3:**
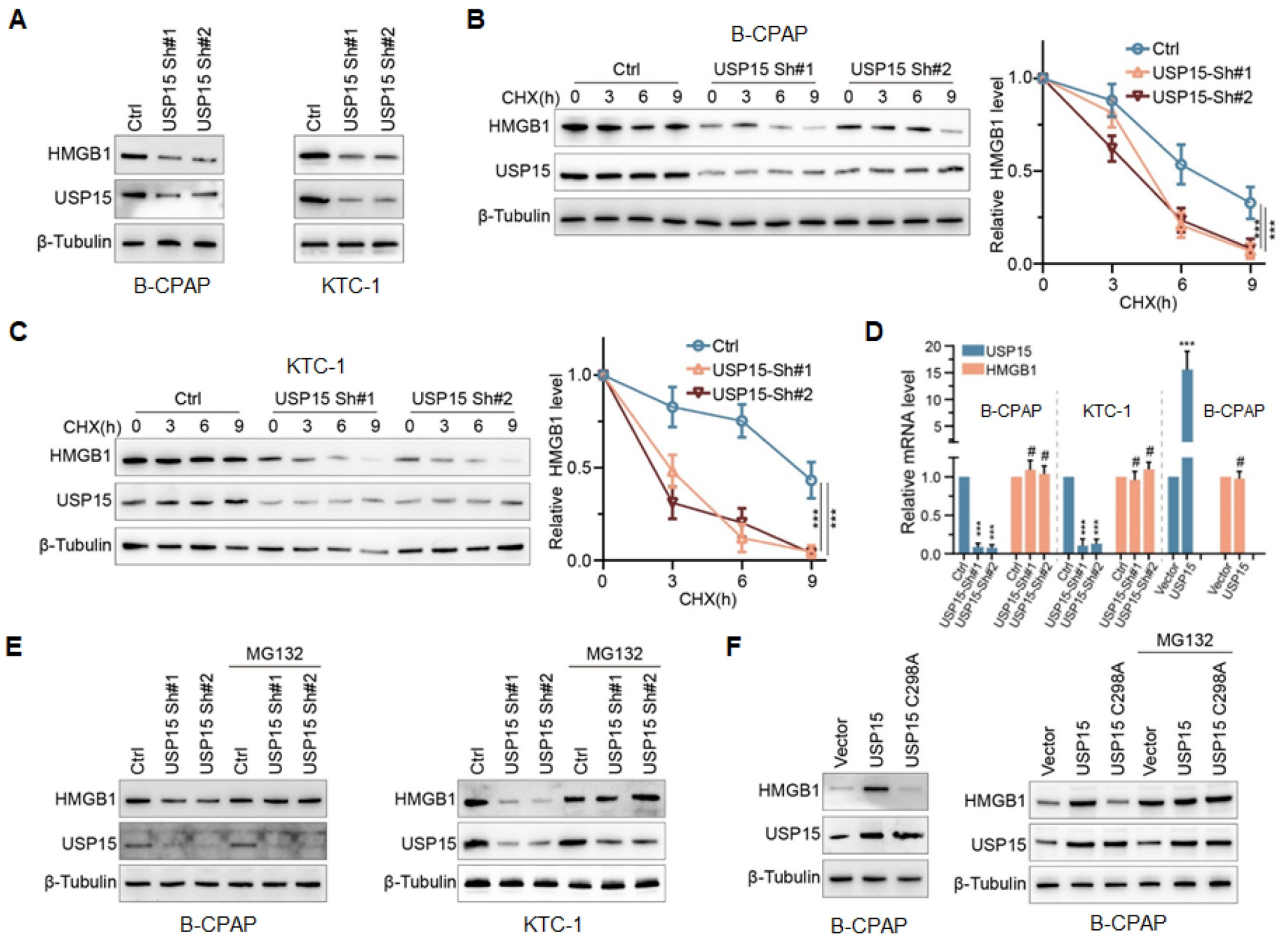
USP15 modulates HMGB1 stability in a DUB(Deubiquitinases)-dependent manner. **A:** The expression levels of HMGB1 protein in B-CPAP cells and KTC-1 cells knocked down USP15. The expression levels of HMGB1 protein in USP15-deficient B-CPAP cells (**B**) and KTC-1 cells (**C**) were treated with CHX (cycloheximide) at different times. **D:** HMGB1 mRNA expression levels in PTC cells with overexpression of USP15 or with low expression of USP15. **E:** The expression levels of HMGB1 protein in USP15-deficient PTC cells treated with the peptide-aldehyde proteasome inhibitor MG132 (carbobenzoxyl-L-leucyl-L-leucyl-L-leucine). **F:** The expression levels of HMGB1 protein in USP15 C298A transfected PTC cells treated with MG132. ***P<0.001.

To determine the effect of USP15 on HMGB1, endogenous USP15 of PTC cells was knocked down. As shown in Figure 3A, the knockdown of USP15 dramatically decreased HMGB1 protein expression in B-CPAP cells and KTC-1 cells compared with that in the Ctrl group. Furthermore, when PTC cells low-expressing USP15 were treated with cycloheximide (CHX), a kind of protein synthesis inhibitor, the half-life of HMGB1 protein was depleted (Figures 3B and 3C) (P<0.001). There were two possible explanations for USP15 in regulating the HMGB1 protein level, which could be either transcriptional regulation or posttranslational regulation. Results of qRT-PCR analysis indicated that HMGB1 mRNA level was not changed upon USP15 depletion compared with that in the Ctrl group (Figure 3D). However, Figure 3D also shows that the level of HMGB1 mRNA did not increase when USP15 was overexpressed in B-CPAP. These results suggested that USP15 did not regulate HMGB1 protein expression at the transcriptional level. However, the peptide-aldehyde proteasome inhibitor MG132 (carbobenzoxyl-L-leucyl-L-leucyl-L-leucine) could abolish the inhibition effect of HMGB1 protein induced by USP15 depletion (Figure 3E). USP15 regulated HMGB1 in a DUB-dependent manner as the catalytically inactive mutant C298A lost its ability to upregulate HMGB1 (Figure 3F). Above all results demonstrated that USP15 increased HMGB1 stability in a DUB-dependent manner.

### USP15 deubiquitylates HMGB1

**Figure 4:**
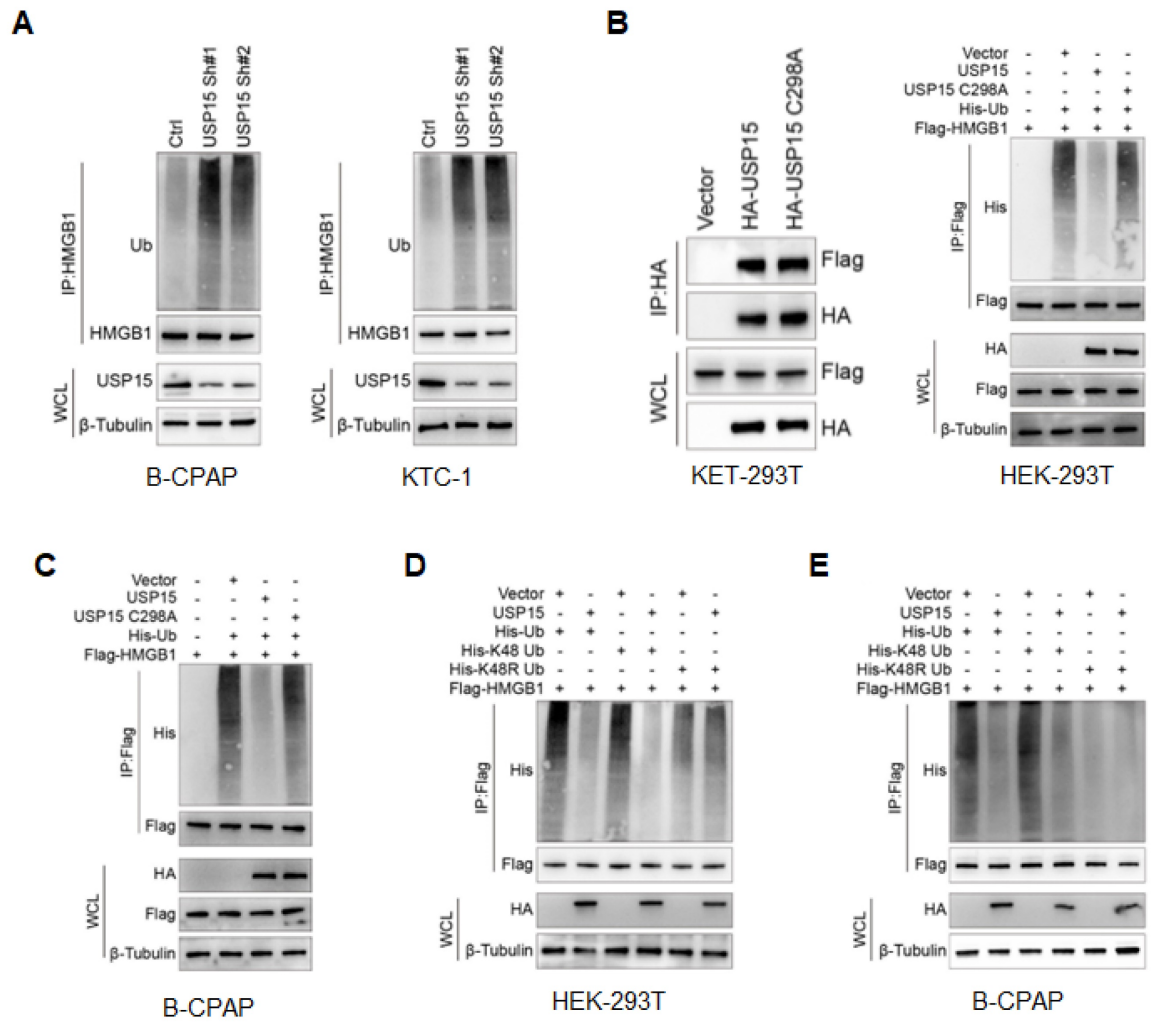
USP15 deubiquitylates HMGB1. **A:** Immunoblotting to detect the ubiquitination of HMGB1 protein in PTC cells transfected with USP15 shRNA. Immunoblotting to detect the ubiquitination of HMGB1 protein in HEK-293T cells (**B**) and B-CPAP cells (**C**) transfected with USP15 and USP15 C298A mutant. Immunoblotting to detect the His-tag in USP 15 overexpression HEK-293T cells (**D**) and B-CPAP cells (**E**) transfected with His-Ub, His-K48 Ub, and His-K48R Ub mutant.

As USP15 was a member of the USP family of DUBs family 25, 37, we went on to determine the possibility that USP15 deubiquitylates HMGB1. The level of polyubiquitin chains on HMGB1 was significantly increased in PTC cells depleted with USP15 (Figure 4A). Conversely, ectopic overexpression of USP15 reduced HMGB1 ubiquitylation in cells, while the C298A mutant of USP15 shut down this function without losing the ability to bind to HMGB1 (Figure 4B). In B-CPAP cells, ubiquitylation assay further confirmed that USP15 directly removed the ubiquitin chain from HMGB1 (Figure 4C). Supporting our former conclusion that the catalytical activity was essential for USP15 to increase HMGB1 stability. It was well known that ubiquitin has several lysine residues (Ub-K6, Ub-K11, Ub-K27, Ub-K29, Ub-K33, Ub-K48, and Ub-K63), which could be used to form distinct linkage types of ubiquitin chains and perform different cellular functions 38. Among them, ubiquitin associated with proteasome degradation was converted to K48. Our results indicated that USP15 was able to efficiently remove the K48-linked ubiquitin chain from HMGB1 (Figures 4D and 4E). Taken together, USP15 was a specific DUB responsible for HMGB1 deubiquitination and stabilization.

### USP15 promotes PTC progression via HMGB1

**Figure 5:**
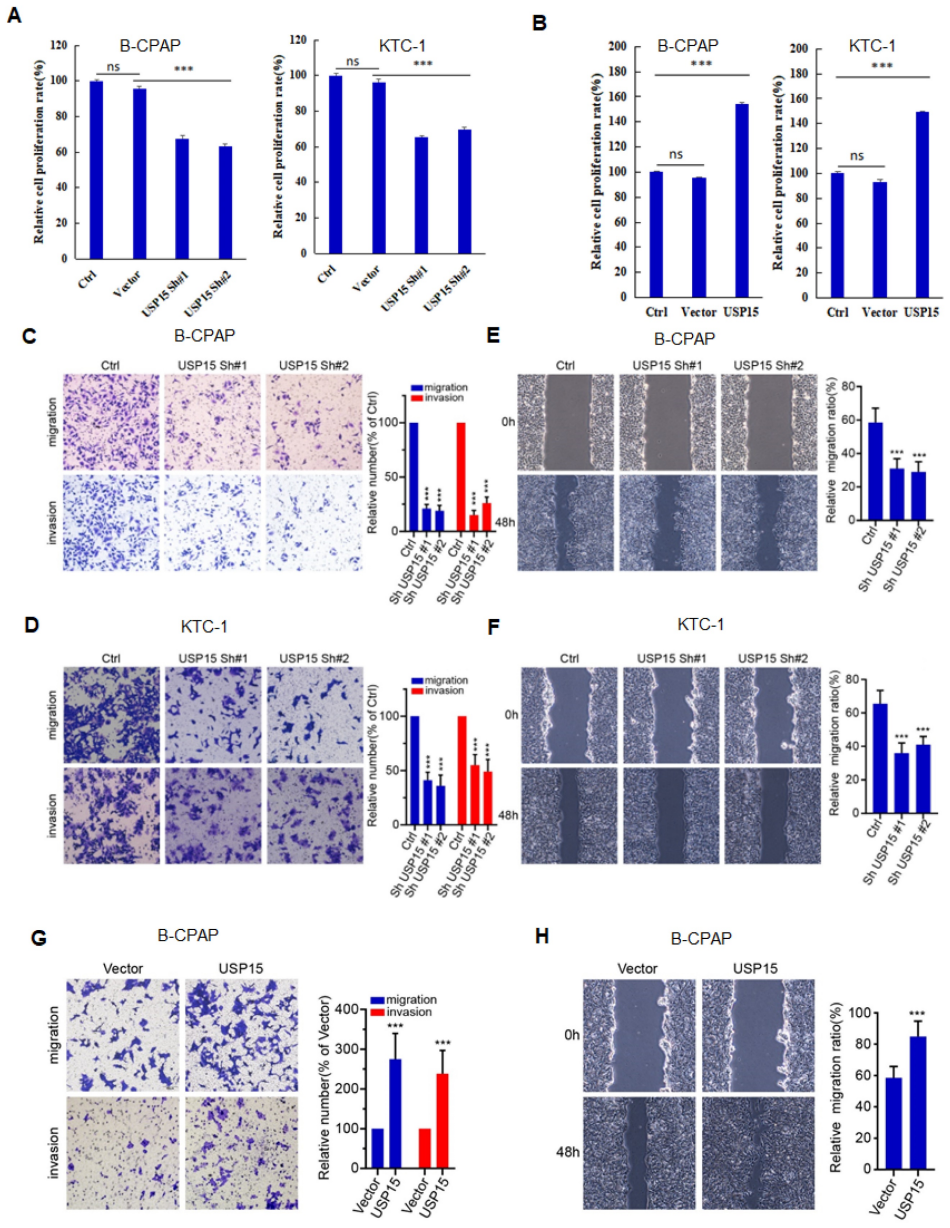
USP15 promotes PTC progression. **A:** Relative cell proliferation rate of PTC cells transfected with USP15 shRNA. **B:** Relative cell proliferation rate of PTC cells transfected with USP15. The results of migration and invasion of B-CPAC cells (**C**) and KTC-1 cells (**D**) transfected with USP15 shRNA. The results of wound healing of B-CPAC cells (**E**) and KTC-1 cells (**F**) transfected with USP15 shRNA. The results of transwell invasion (**G**) and wound healing (**H**) of B-CPAP cells transfected with USP15. ***P<0.001 and ns: no significance.

**Figure 6:**
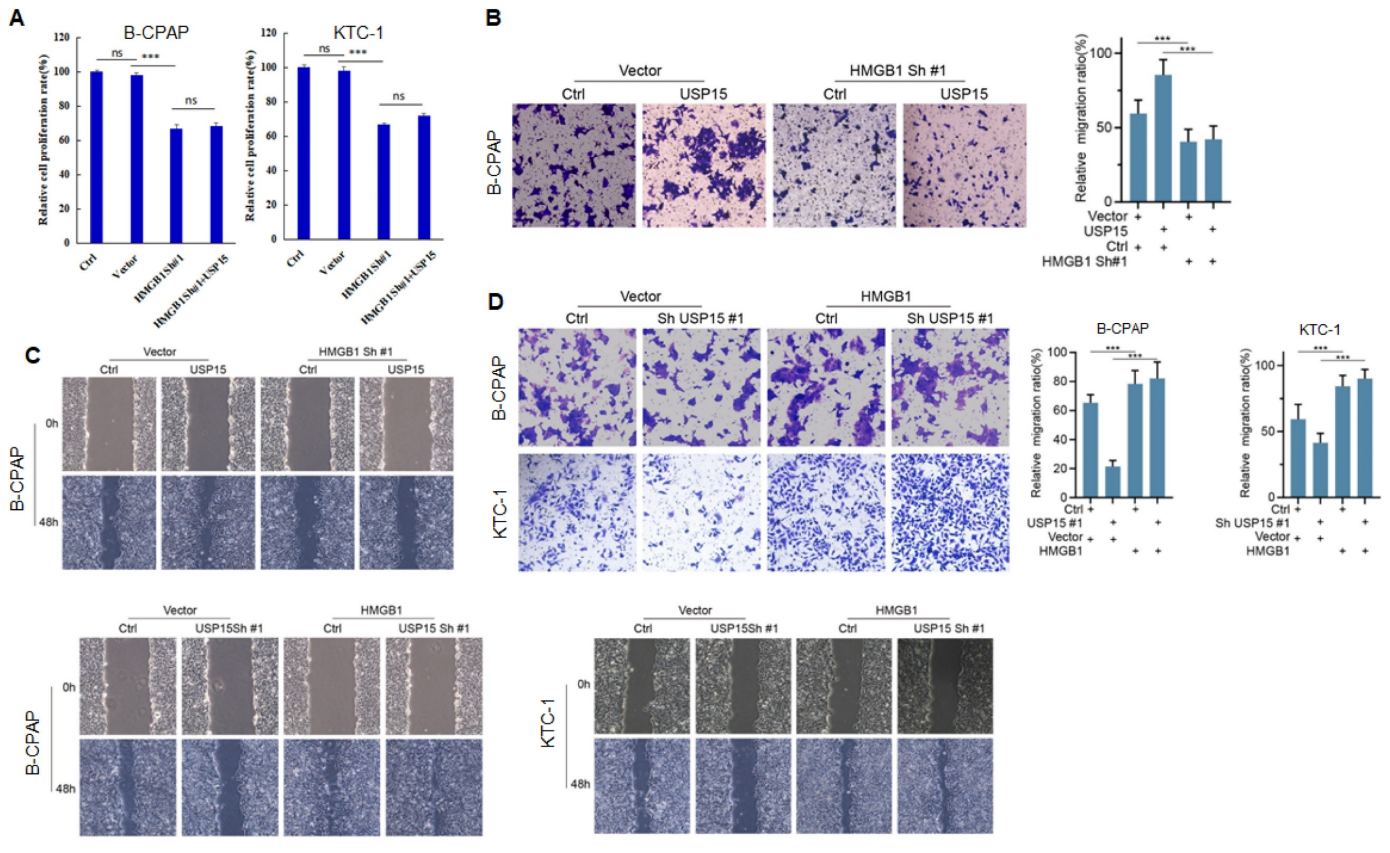
USP15 promotes PTC progression via HMGB1.** A:** Relative cell proliferation rate of PTC cells co-transfected with HMGB1 shRNA and USP15. The results of migration (**B**) and wound healing (**C**) of B-CPAC cells co-transfected with HMGB1 shRNA and USP15. The results of migration (**D**) and wound healing (**E**) of B-CPAC cells co-transfected with HMGB1 and USP15 shRNA. ***P<0.001 and ns: no significance.

We further investigated the function of USP15 in two PTC cell lines (KTC-1 and B-CPAP). USP15 depletion significantly decreased cell proliferation rate (Figure 5A) (PTC cell *VS* Ctrl and Vector, P<0.001). In contrast, overexpression of USP15 promoted cell proliferation (Figure 5B) (PTC cell *VS* Ctrl and Vector, P<0.001). Furthermore, the knockdown of USP15 could also reduce the migration and invasion capacity of PTC cells as evaluated by transwell invasion assays (Figures 5C and 5D) (PTC cell *VS* Ctrl, P<0.001) and wound healing (Figures 5E and 5F) (PTC cell *VS* Ctrl, P<0.001), whereas this phenomenon was reversed in USP15 overexpressed B-CPAP cells (Figures 5G and 5H) (PTC cell *VS* Vector P<0.001). To determine whether the functions of USP15 in regulating cell proliferation, migration, and invasion through the effects of HMGB1, we performed rescue experiments by overexpressing HMGB1 in USP15 knockdown cells or by knockdown HMGB1 in USP15 overexpression cells. Increased HMGB1 expression induced cell proliferation (Figure 1B), while depletion of HMGB1 (Figure S1) inhibited cell proliferation, even though USP15 was overexpressed (Figure 6A). Furthermore, we further verified that overexpression of USP15 could not improve the migration and invasion ability of PTC cells when HMGB1 was knocked down (Figures 6B and 6C). Transwell migration and wound healing assays indicated that the re-expression of HMGB1 largely rescued the migration and invasion capacity of PTC cells (Figures 6D and 6E). Taken together, these results indicated that USP15 promoted PTC progression through HMGB1.

## Discussion

PTC is considered to be one of the most common TCs, prone to in-situ recurrence and distant metastasis 1. Understanding the genetic and molecular mechanisms of PTC will bring new hope for targeted therapy of the disease 13. HMGB1 is a highly conserved nuclear protein, which can participate in the development of cancer cells by regulating chromatin remodeling, gene transcription, and DNA replication 39. Furthermore, HMGB1 stability is closely related to its ubiquitination 40, 41. Yao *et al.* 42 demonstrated that KDM4D transcriptionally activates SYVN1 expressions via H3K9me3 demethylation at the promoter region, thereby triggering the ubiquitin-dependent degradation of HMGB1. Therefore, ubiquitination, an important post-translational modification, is essential for cell homeostasis 43. It should be noted that the ubiquitination of cellular proteins is a reversible and dynamic process, constantly being ubiquitinated and deubiquitinated. This process is precisely planned and executed by ubiquitin ligases and DUBs.

In this study, we demonstrated that HMGB1 was able to promote the development of PTC. The interaction between USP15 and HMGB1 was identified by LC-ESL-LTQ-Orbitrap-MS and immunoprecipitation. Fukagai *et al.* 44 had shown that USP15 could stabilize and activate Nrf1 in the nucleus through deubiquitination, and knockdown of USP15 reduced the expression of NRF1-induced proteasome, to participate in maintaining protein homeostasis at the transcriptional level. In contrast, USP15 did not affect the mRNA expression of HMGB1, but HMGB1 protein expression was significantly decreased in PTC cells after USP15 knockdown expression, indicating that the level of HMGB1 protein was regulated by DUB USP15. In addition, HMGB1 was deubiquitinized by USP15 but not by inactive USP15 C298A mutant or USP15 shRNA, suggesting that HMGB1 deubiquitination is regulated by USP15. K48 polyubiquitination at ubiquitin usually resulted in proteasome degradation 45. Studies have shown that USP15 knockdown induced downregulation of estrogen receptor alpha protein by promoting its K48-related ubiquitination, which is necessary for proliferation inhibition of breast cancer cells 46. Similarly, when USP15 was overexpressed, the HGMB1 protein level decreased due to the reduction of its associated ubiquitination by transfecting the K48R mutant into PTC cells 47. Moreover, the development of malignant tumors, including invasion and metastasis, depended on the integrity of the basement membrane and extracellular matrix. A previous study showed that HMGB1 could bind to RAGE, activate the MAPK signaling pathway, and then cause the activation of matrix proteases MMP9 and MMP2, degraded extracellular matrix, and promote tumor invasion and metastasis 48. Based on the previous study, we speculated that USP15 deubiquitinated HMGB1 protein via K48, thereby improving the stability of HMGB1 in PTC cells. Despite conducting in-depth research on the relationship between USP15 and HMGB1, it is worth noting that this study still has some limitations. 1) Limited Clinical Validation: While our study elucidates the role of HMGB1 and USP15 in PTC development through in vitro experiments, the clinical relevance of these findings remains to be fully established. Further investigations involving clinical samples from PTC patients are essential to determine whether the observed molecular mechanisms hold in real-world scenarios and whether targeting HMGB1 and USP15 could lead to improved clinical outcomes. 2) Simplification of Molecular Interactions: Our study presents a simplified model of the interactions between HMGB1, USP15, and PTC development. However, biological systems are inherently complex, and multiple factors, signaling pathways, and feedback mechanisms might influence the observed outcomes. The extent to which our findings accurately reflect the intricacies of these interactions warrants further investigation. 3) Lack of In Vivo Validation:

While our study provides insights into the cellular mechanisms involved, it predominantly relies on in vitro experiments. The absence of in vivo validation using animal models or patient-derived xenografts limits our ability to fully understand how the identified molecular interactions translate to the complex tumor microenvironment in vivo. The inclusion of in vivo experiments would be crucial for confirming the clinical potential of targeting HMGB1 and USP15 for PTC therapy.

Our study demonstrated that the proliferation, invasion, and metastasis of PTC cells with low expression of USP15 were significantly inhibited, while the results of overexpression of USP15 were the opposite. However, even if USP15 was overexpressed in PTC cells, cell proliferation, migration, and infiltration were still hindered when HMGB1 was knocked down. This hindrance to PTC development would disappear after increasing the HMGB1 expression level. It was not difficult to find that USP15 was a potent DUB responsible for HMGB1 and USP15 promoted PTC progression by K48-linked deubiquitinating and stabilizing HMGB1. Our findings provide new insight into the roles of USP15 in the progression of PTC, regulating the activity of USP15 or regulating its gene expression level may be a promising strategy for treating PTC.

## Supplementary Material

Supplementary figure and tables.
