# Supplementary material for: USP15 promotes the progression of papillary thyroid cancer by regulating HMGB1 stability through its deubiquitination
Source: J Cancer. 2024 Mar 11;15(9):2561–72. doi: 10.7150/jca.92386 (PMC10988300; doi:10.7150/jca.92386)
Supplement: Supplementary file 1 — Supplementary figure and tables. [file jcav15p2561s1.pdf]

Figure S1. The protein expression (A) and mRNA transcription (B) levels of HMGB1 in HEK-293T cells transfected with HMGB1 ShRNA. ***P<0.001.

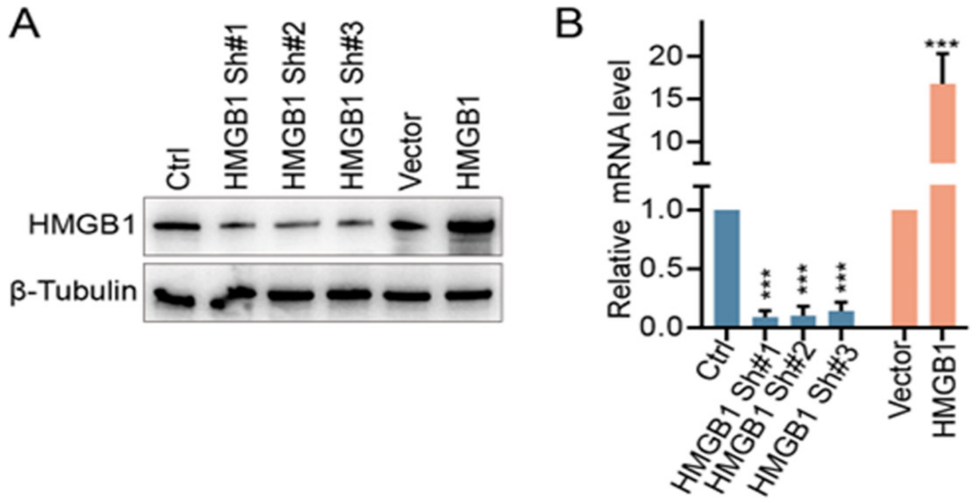

Table S1 The enriched protein peptides were analyzed by mass spectrometry

Accession	Protein names	Gene names	MW [kDa]	Protein score	Sequence coverage (%)	# Unique Peptides	# Peptides	# PSMs
P09429	High mobility group protein B1	HMGB1	24.9	1072	24	26	29	32
Q00839	Heterogeneous nuclear ribonucleoprotein U	HNRNPU	90.5	879	26	20	23	25
Q9Y4E8	Ubiquitin carboxyl-terminal hydrolase 15	USP15	112.4	571	30	20	21	20
Q9NZB2	Constitutive coactivator of PPAR-gamma-like protein 1	FAM120A	121.8	738	26	19	20	21
P11177	Pyruvate dehydrogenase E1 component subunit beta, mitochondrial	PDHB	39.2	1111	50	17	18	33
Q96JN8	Neuralized-like protein 4	NEURL4	166.8	331	13	15	15	13
P23396	40S ribosomal protein S3	RPS3	26.7	375	47	14	19	13
Q96PK6	RNA-binding protein 14	RBM14	69.4	395	19	13	15	13
P62241	40S ribosomal protein S8	RPS8	24.2	295	54	12	13	11
Q12906	Interleukin enhancer-binding factor 3	ILF3	95.3	301	12	11	16	10
O00567	Nucleolar protein 56	NOP56	66	441	29	11	16	12
P27635	60S ribosomal protein L10	RPL10	24.6	224	38	11	15	8
P62424	60S ribosomal protein L7a	RPL7A	30	365	26	10	11	10
P11940	Polyadenylate-binding protein 1	PABPC1	70.6	565	24	10	11	18
Q15424	Scaffold attachment factor B1	SAFB	102.6	99	7	10	11	7
P62906	60S ribosomal protein L10a	RPL10A	24.8	263	41	10	14	12
P62249	40S ribosomal protein S16	RPS16	16.4	182	43	9	14	9
P46782	40S ribosomal protein S5	RPS5	22.9	331	34	9	9	8
Q99459	Cell division cycle 5-like protein	CDC5L	92.2	342	14	9	11	8
P51991	Heterogeneous nuclear ribonucleoprotein A3	HNRNPA3	39.6	199	19	9	13	10
P40429	60S ribosomal protein L13a	RPL13A	23.6	200	26	9	10	7
P31943	Heterogeneous nuclear ribonucleoprotein H	HNRNPH1	49.2	884	37	9	13	21
Q9UQE7	Structural maintenance of chromosomes protein 3	SMC3	141.5	160	8	9	13	7
Q9HAU5	Regulator of nonsense transcripts 2	UPF2	147.7	161	6	9	14	6

P60842	Eukaryotic initiation factor 4A-I	EIF4A1	46.1	187	17	8	12	6
Q96B26	Exosome complex component RRP43	EXOSC8	30	157	27	8	12	7
P46087	Probable 28S rRNA (cytosine(4447)-C(5))-methyltransferase	NOP2	89.2	279	14	8	12	8
Q15029	116 kDa U5 small nuclear ribonucleoprotein component	EFTUD2	109.4	158	10	8	10	6
Q9NY93	Probable ATP-dependent RNA helicase DDX56	DDX56	61.6	203	14	8	9	5
Q9HCD5	Nuclear receptor coactivator 5	NCOA5	65.5	245	17	8	8	7
P61513	60S ribosomal protein L37a	RPL37A	10.3	139	51	7	7	5
P52292	Importin subunit alpha-1	KPNA2	57.8	320	22	7	11	10
Q9NZI8	Insulin-like growth factor 2 mRNA-binding protein 1	IGF2BP1	63.4	361	15	7	7	7
Q9NX05	Constitutive coactivator of PPAR-gamma-like protein 2	FAM120C	120.5	233	10	7	8	8
P49207	60S ribosomal protein L34	RPL34	13.3	79	29	7	9	4
P55060	Exportin-2	CSE1L	110.3	207	6	7	9	6
Q07666	KH domain-containing, RNA-binding, signal transduction-associated protein 1	KHDRBS1	48.2	200	12	7	7	7
Q03701	CCAAT/enhancer-binding protein zeta	CEBPZ	120.9	87	7	7	9	4
O43684	Mitotic checkpoint protein BUB3	BUB3	37.1	129	13	7	12	5
O43791	Speckle-type POZ protein	SPOP	42.1	207	12	7	10	3
Q5JTH9	RRP12-like protein	RRP12	143.6	92	5	7	11	3
Q7Z2W4	Zinc finger CCCH-type antiviral protein 1	ZC3HAV1	101.4	72	5	7	10	3
Q96SB4	SRSF protein kinase 1	SRPK1	74.3	63	8	7	9	5
P62081	40S ribosomal protein S7	RPS7	22.1	260	42	7	9	10
Q9BU76	Multiple myeloma tumor-associated protein 2	MMTAG2	29.4	62	11	6	11	2
P22234	Multifunctional protein ADE2	PAICS	47	75	12	6	11	4
Q13823	Nucleolar GTP-binding protein 2	GNL2	83.6	242	9	6	9	5
P51114	Fragile X mental retardation syndrome-related protein 1	FXR1	69.7	171	16	6	7	7
P34932	Heat shock 70 kDa protein 4	HSPA4	94.3	170	6	6	8	3
P25205	DNA replication licensing factor MCM3	MCM3	90.9	131	8	6	7	5
P14866	Heterogeneous nuclear ribonucleoprotein L	HNRNPL	64.1	82	6	6	9	3
P63173	60S ribosomal protein L38	RPL38	8.2	203	38	6	8	7
P18077	60S ribosomal protein L35a	RPL35A	12.5	50	18	6	6	2
P62266	40S ribosomal protein S23	RPS23	15.8	182	36	6	7	5
Q8WXX5	DnaJ homolog subfamily C member 9	DNAJC9	29.9	128	13	6	7	4
Q15269	Periodic tryptophan protein 2 homolog	PWP2	102.4	37	3	6	8	4
Q08170	Serine/arginine-rich splicing factor 4	SRSF4	56.6	139	10	6	10	6
Q8NHQ9	ATP-dependent RNA helicase DDX55	DDX55	68.5	77	10	6	10	4
P00558	Phosphoglycerate kinase 1	PGK1	44.6	117	12	6	6	6
Q14739	Delta(14)-sterol reductase LBR	LBR	70.7	50	5	6	7	2

Q13310	Polyadenylate-binding protein 4	PABPC4	70.7	423	22	6	7	10
Q13347	Eukaryotic translation initiation factor 3 subunit I	EIF3I	36.5	60	7	6	9	3
P06733	Alpha-enolase	ENO1	47.1	172	16	6	8	6
P62854	40S ribosomal protein S26	RPS26	13	94	37	6	6	6
Q9H814	Phosphorylated adapter RNA export protein	PHAX	44.4	69	7	5	8	3
Q6ZN17	Protein lin-28 homolog B	LIN28B	27.1	28	9	5	10	1
P41252	Isoleucine--tRNA ligase, cytoplasmic	IARS1	144.4	82	1	5	7	2
Q9P035	Very-long-chain (3R)-3-hydroxyacyl-CoA dehydratase 3	HACD3	43.1	128	11	5	6	3
O95980	Reversion-inducing cysteine-rich protein with Kazal motifs	RECK	106.4	26	1	5	8	1
P14635	G2/mitotic-specific cyclin-B1	CCNB1	48.3	30	6	5	7	1
Q9UK59	Lariat debranching enzyme	DBR1	61.5	30	5	5	9	2
Q96ME7	Zinc finger protein 512	ZNF512	64.6	21	3	5	9	1
O60231	Pre-mRNA-splicing factor ATP-dependent RNA helicase DHX16	DHX16	119.2	49	2	5	9	2
Q3SXM5	Inactive hydroxysteroid dehydrogenase-like protein 1	HSDL1	37	67	5	5	10	3
P17987	T-complex protein 1 subunit alpha	TCP1	60.3	43	3	5	6	1
Q9NWH9	SAFB-like transcription modulator	SLTM	117.1	70	4	5	9	1
Q86UK5	Limbin	EVC2	147.9	28	1	5	5	3
Q8NEY8	Periphilin-1	PPHLN1	52.7	34	3	5	10	2
Q9GZR7	ATP-dependent RNA helicase DDX24	DDX24	96.3	176	7	5	5	4
A6NHL2	Tubulin alpha chain-like 3	TUBAL3	49.9	191	10	5	7	5
Q86UK7	E3 ubiquitin-protein ligase ZNF598	ZNF598	98.6	35	4	5	10	2
Q02790	Peptidyl-prolyl cis-trans isomerase FKBP4	FKBP4	51.8	122	4	5	7	2
Q96SB8	Structural maintenance of chromosomes protein 6	SMC6	126.2	47	3	5	9	3
Q14683	Structural maintenance of chromosomes protein 1A	SMC1A	143.1	102	5	5	9	5
P61163	Alpha-centractin	ACTR1A	42.6	54	5	5	10	2
P55081	Microfibrillar-associated protein 1	MFAP1	51.9	21	6	5	5	3
O95232	Luc7-like protein 3	LUC7L3	51.4	95	7	5	5	3
Q6UUV7	CREB-regulated transcription coactivator 3	CRTC3	66.9	61	6	5	8	2
P42167	Lamina-associated polypeptide 2, isoforms beta/gamma	TMPO	50.6	42	3	5	10	3
Q709F0	Acyl-CoA dehydrogenase family member 11	ACAD11	87.2	26	3	5	8	3
Q92804	TATA-binding protein-associated factor 2N	TAF15	61.8	217	17	5	10	6
P80404	4-aminobutyrate aminotransferase, mitochondrial	ABAT	56.4	26	4	5	5	2
P22314	Ubiquitin-like modifier-activating enzyme 1	UBA1	117.8	84	7	5	7	4
Q99623	Prohibitin-2	PHB2	33.3	41	6	5	6	3

Q15120	[Pyruvate dehydrogenase (acetyl-transferring)] kinase isozyme 3, mitochondrial	PDK3	46.9	43	4	5	6	2
P45974	Ubiquitin carboxyl-terminal hydrolase 5	USP5	95.7	88	3	5	7	2
Q14320	Protein FAM50A	FAM50A	40.2	31	3	5	9	2
Q96QV6	Histone H2A type 1-A	H2AC1	14.2	29	19	5	8	1
Q2NL82	Pre-rRNA-processing protein TSR1 homolog	TSR1	91.8	105	4	5	5	4
Q99714	3-hydroxyacyl-CoA dehydrogenase type-2	HSD17B10	26.9	28	7	5	10	1
P29372	DNA-3-methyladenine glycosylase	MPG	32.8	50	7	5	6	2
Q13838	Spliceosome RNA helicase DDX39B	DDX39B	49	93	12	5	6	6
P31040	Succinate dehydrogenase [ubiquinone] flavoprotein subunit, mitochondrial	SDHA	72.6	70	3	5	7	1
Q9UKX7	Nuclear pore complex protein Nup50	NUP50	50.1	37	5	5	8	3
Q9Y314	Nitric oxide synthase-interacting protein	NOSIP	33.2	25	7	5	5	3
Q6UXN9	WD repeat-containing protein 82	WDR82	35.1	63	7	5	8	2
Q14974	Importin subunit beta-1	KPNB1	97.1	32	3	5	5	3
Q9ULX6	A-kinase anchor protein 8-like	AKAP8L	71.6	40	2	5	7	1
Q9BZZ5	Apoptosis inhibitor 5	API5	59	220	10	5	5	5
Q7Z478	ATP-dependent RNA helicase DHX29	DHX29	155.1	150	5	5	6	4
Q53GQ0	Very-long-chain 3-oxoacyl-CoA reductase	HSD17B12	34.3	50	4	5	6	3
Q9BRJ6	Uncharacterized protein C7orf50	C7orf50	22.1	45	8	5	7	3
Q6DKI1	60S ribosomal protein L7-like 1	RPL7L1	29.7	24	4	5	9	2
Q8NBS9	Thioredoxin domain-containing protein 5	TXNDC5	47.6	26	7	5	5	1
Q14240	Eukaryotic initiation factor 4A-II	EIF4A2	46.4	347	18	5	9	12
P32119	Peroxiredoxin-2	PRDX2	21.9	46	11	5	8	3
Q15050	Ribosome biogenesis regulatory protein homolog	RRS1	41.2	95	10	4	8	3
Q8IY37	Probable ATP-dependent RNA helicase DHX37	DHX37	129.5	47	3	4	6	3
O60841	Eukaryotic translation initiation factor 5B	EIF5B	138.7	33	1	4	5	3
P52597	Heterogeneous nuclear ribonucleoprotein F	HNRNPF	45.6	281	26	4	5	10
Q86XZ4	Spermatogenesis-associated serine-rich protein 2	SPATS2	59.5	71	6	4	6	3
P53007	Tricarboxylate transport protein, mitochondrial	SLC25A1	34	27	3	4	9	3
P01706	Immunoglobulin lambda variable 2-11	IGLV2-11	12.6	42	9	4	8	3
P13804	Electron transfer flavoprotein subunit alpha, mitochondrial	ETFA	35.1	54	5	4	8	3
P00505	Aspartate aminotransferase, mitochondrial	GOT2	47.5	27	3	4	5	3
Q9BYN8	28S ribosomal protein S26, mitochondrial	MRPS26	24.2	28	9	4	8	3
Q13164	Mitogen-activated protein kinase 7	MAPK7	88.3	27	1	4	6	2
P17980	26S proteasome regulatory subunit 6A	PSMC3	49.2	40	5	4	9	1
Q9UKK9	ADP-sugar pyrophosphatase	NUDT5	24.3	27	5	4	7	3

Q9NYL2	Mitogen-activated protein kinase kinase kinase 20	MAP3K20	91.1	74	2	4	5	1
P60866	40S ribosomal protein S20	RPS20	13.4	95	21	4	4	2
P56537	Eukaryotic translation initiation factor 6	EIF6	26.6	29	6	4	8	2
A6NKL6	Transmembrane protein 200C	TMEM200C	63.9	26	1	4	7	3
P42704	Leucine-rich PPR motif-containing protein, mitochondrial	LRPPRC	157.8	94	3	4	8	3
Q13868	Exosome complex component RRP4	EXOSC2	32.8	237	24	4	8	5
P52926	High mobility group protein HMGI-C	HMGA2	11.8	22	22	4	6	2
Q9HAV4	Exportin-5	XPO5	136.2	29	1	4	8	3
Q92900	Regulator of nonsense transcripts 1	UPF1	124.3	48	1	4	8	2
Q7Z333	Probable helicase senataxin	SETX	302.7	24	0	4	6	1
P54819	Adenylate kinase 2, mitochondrial	AK2	26.5	70	10	4	8	3
A6NHR9	Structural maintenance of chromosomes flexible hinge domain-containing protein 1	SMCHD1	226.2	46	3	4	4	2
Q9ULX3	RNA-binding protein NOB1	NOB1	46.6	34	4	4	8	3
Q9Y421	Protein FAM32A	FAM32A	13.2	30	9	4	7	1
Q86SU0	Immunoglobulin-like domain-containing receptor 1	ILDR1	62.8	29	2	4	4	3
Q86YQ8	Copine-8	CPNE8	63.1	54	2	4	7	2
Q86VP6	Cullin-associated NEDD8-dissociated protein 1	CAND1	136.3	33	1	4	9	2
Q9BQ52	Zinc phosphodiesterase ELAC protein 2	ELAC2	92.2	29	3	4	5	3
Q9Y520	Protein PRRC2C	PRRC2C	316.7	23	1	4	4	3
Q13247	Serine/arginine-rich splicing factor 6	SRSF6	39.6	51	4	4	9	2
Q5C9Z4	Nucleolar MIF4G domain-containing protein 1	NOM1	96.2	81	4	4	9	1
P42677	40S ribosomal protein S27	RPS27	9.5	90	32	4	6	5
Q9BY44	Eukaryotic translation initiation factor 2A	EIF2A	64.9	66	5	4	9	3
P52732	Kinesin-like protein KIF11	KIF11	119.1	31	3	4	9	3
Q96CU9	FAD-dependent oxidoreductase domain-containing protein 1	FOXRED1	53.8	40	4	4	7	1
Q9BPU6	Dihydropyrimidinase-related protein 5	DPYSL5	61.4	31	3	4	7	2
P62195	26S proteasome regulatory subunit 8	PSMC5	45.6	65	3	4	8	1
O75367	Core histone macro-H2A.1	MACROH2A1	39.6	43	5	4	5	3
P08195	4F2 cell-surface antigen heavy chain	SLC3A2	68	46	5	4	8	4
A0A0C4DH72	Immunoglobulin kappa variable 1-6	IGKV1-6	12.7	26	14	4	9	3
Q9NPD3	Exosome complex component RRP41	EXOSC4	26.4	93	11	4	5	2
P04196	Histidine-rich glycoprotein	HRG	59.5	44	2	4	4	2
Q9HCM2	Plexin-A4	PLXNA4	212.3	23	2	4	9	3
P24539	ATP synthase F(0) complex subunit B1, mitochondrial	ATP5PB	28.9	29	3	4	9	2
Q9NQG5	Regulation of nuclear pre-mRNA domain-containing protein 1B	RPRD1B	36.9	85	12	3	6	4
P69905	Hemoglobin subunit alpha	HBA1	15.2	70	8	3	5	2

Q5QNW6	Histone H2B type 2-F	H2BC18	13.9	401	37	3	6	10
Q2TAY7	WD40 repeat-containing protein SMU1	SMU1	57.5	88	5	3	7	4
Q8NAB2	Kelch repeat and BTB domain-containing protein 3	KBTBD3	69.8	22	1	3	6	2
P61326	Protein mago nashi homolog	MAGOH	17.2	33	8	3	4	2
P11766	Alcohol dehydrogenase class-3	ADH5	39.7	23	2	3	7	2
A0A075B6S5	Immunoglobulin kappa variable 1-27	IGKV1-27	12.7	49	14	3	4	3
Q8NC24	RELT-like protein 2	RELL2	32.4	24	2	3	7	2
O95373	Importin-7	IPO7	119.4	53	1	3	3	2
P06730	Eukaryotic translation initiation factor 4E	EIF4E	25.1	24	7	3	5	1
Q92615	La-related protein 4B	LARP4B	80.5	20	1	3	6	1
Q14152	Eukaryotic translation initiation factor 3 subunit A	EIF3A	166.5	68	3	3	8	4
Q13895	Bystin	BYSL	49.6	65	5	3	4	3
Q13765	Nascent polypeptide-associated complex subunit alpha	NACA	23.4	33	8	3	4	3
A0A0B4J2D9	Immunoglobulin kappa variable 1D-13	IGKV1D-13	12.6	35	15	3	8	1
Q9H361	Polyadenylate-binding protein 3	PABPC3	70	970	26	3	8	28
Q92545	Transmembrane protein 131	TMEM131	205	24	0	3	5	2
Q16891	MICOS complex subunit MIC60	IMMT	83.6	47	4	3	8	2
P29401	Transketolase	TKT	67.8	56	3	3	6	2
Q02218	2-oxoglutarate dehydrogenase, mitochondrial	OGDH	115.9	73	3	3	6	5
Q7LGA3	Heparan sulfate 2-O-sulfotransferase 1	HS2ST1	41.9	26	3	3	4	3
Q16637	Survival motor neuron protein	SMN1	31.8	77	7	3	4	5
P55010	Eukaryotic translation initiation factor 5	EIF5	49.2	25	5	3	7	2
P55786	Puromycin-sensitive aminopeptidase	NPEPPS	103.2	49	1	3	8	3
Q9BRZ2	E3 ubiquitin-protein ligase TRIM56	TRIM56	81.4	26	5	3	4	1
P04004	Vitronectin	VTN	54.3	49	3	3	8	1
Q14331	Protein FRG1	FRG1	29.2	55	5	3	4	2
Q8WXF0	Serine/arginine-rich splicing factor 12	SRSF12	30.5	22	4	3	6	3
P63208	S-phase kinase-associated protein 1	SKP1	18.6	45	7	3	6	1
P11388	DNA topoisomerase 2-alpha	TOP2A	174.3	39	2	3	6	1
Q5TBA9	Protein furry homolog	FRY	338.7	28	1	3	7	2
P12956	X-ray repair cross-complementing protein 6	XRCC6	69.8	136	8	3	4	4
P62826	GTP-binding nuclear protein Ran	RAN	24.4	35	8	3	8	1
P14174	Macrophage migration inhibitory factor	MIF	12.5	31	11	3	4	3
Q71RC2	La-related protein 4	LARP4	80.5	78	3	3	5	1
P53618	Coatomer subunit beta	COPB1	107.1	41	1	3	6	3
Q9Y277	Voltage-dependent anion-selective channel protein 3	VDAC3	30.6	46	4	3	7	3
Q13263	Transcription intermediary factor 1-beta	TRIM28	88.5	87	4	3	3	4

P17858	ATP-dependent 6-phosphofructokinase, liver type	PFKL	85	46	3	3	5	1
Q9BW92	Threonine--tRNA ligase, mitochondrial	TARS2	81	29	3	3	7	2
O95163	Elongator complex protein 1	ELP1	150.2	24	2	3	6	1
P09429	High mobility group protein B1	HMGB1	24.9	25	9	3	3	1
Q86TB9	Protein PAT1 homolog 1	PATL1	86.8	38	2	3	6	3
P48634	Protein PRRC2A	PRRC2A	228.7	45	2	3	5	3
Q8N511	Transmembrane protein 199	TMEM199	23.1	29	6	3	4	1
P17612	cAMP-dependent protein kinase catalytic subunit alpha	PRKACA	40.6	26	4	3	4	1
P49790	Nuclear pore complex protein Nup153	NUP153	153.8	110	6	3	6	4
P13667	Protein disulfide-isomerase A4	PDIA4	72.9	32	3	3	8	2
P33992	DNA replication licensing factor MCM5	MCM5	82.2	112	7	3	8	5
P30153	Serine/threonine-protein phosphatase 2A 65 kDa regulatory subunit A alpha isoform	PPP2R1A	65.3	23	4	3	6	2
P31948	Stress-induced-phosphoprotein 1	STIP1	62.6	32	2	3	7	1
P49643	DNA primase large subunit	PRIM2	58.8	38	4	2	2	3
Q13610	Periodic tryptophan protein 1 homolog	PWP1	55.8	46	5	2	7	3
O60281	Zinc finger protein 292	ZNF292	304.6	31	2	2	2	2
P12004	Proliferating cell nuclear antigen	PCNA	28.8	75	9	2	4	4
O75494	Serine/arginine-rich splicing factor 10	SRSF10	31.3	45	6	2	5	1
Q14247	Src substrate cortactin	CTTN	61.5	40	2	2	6	3
P61962	DDB1- and CUL4-associated factor 7	DCAF7	38.9	27	3	2	5	2
Q8WXF1	Paraspeckle component 1	PSPC1	58.7	35	6	2	6	3
A0A075B6I1	Immunoglobulin lambda variable 4-60	IGLV4-60	13	51	8	2	4	2
O14980	Exportin-1	XPO1	123.3	22	1	2	3	2
Q92979	Ribosomal RNA small subunit methyltransferase NEP1	EMG1	26.7	41	9	2	7	2
P52756	RNA-binding protein 5	RBM5	92.1	24	4	2	4	1
P09211	Glutathione S-transferase P	GSTP1	23.3	67	17	2	5	2
P52907	F-actin-capping protein subunit alpha-1	CAPZA1	32.9	21	6	2	4	3
Q9Y3Z3	Deoxynucleoside triphosphate triphosphohydrolase SAMHD1	SAMHD1	72.2	42	3	2	7	3
Q96QU6	1-aminocyclopropane-1-carboxylate synthase-like protein 1	ACCS	57.3	29	4	2	7	3
Q52LJ0	Protein FAM98B	FAM98B	45.5	117	11	2	3	4
Q9NUD5	Zinc finger CCHC domain-containing protein 3	ZCCHC3	43.5	51	6	2	2	3
P27824	Calnexin	CANX	67.5	70	5	2	5	1
P35579	Myosin-9	MYH9	226.4	87	3	2	6	3
Q3B820	Protein FAM161A	FAM161A	76.7	22	1	2	4	3
Q8NG50	RAD52 motif-containing protein 1	RDM1	31.9	27	3	2	5	3
Q969Q0	60S ribosomal protein L36a-like	RPL36AL	12.5	54	10	2	5	4

O00303	Eukaryotic translation initiation factor 3 subunit F	EIF3F	37.5	100	9	2	5	2
O00139	Kinesin-like protein KIF2A	KIF2A	79.9	110	3	2	2	4
Q96DA6	Mitochondrial import inner membrane translocase subunit TIM14	DNAJC19	12.5	95	18	2	6	2
Q9Y3D9	28S ribosomal protein S23, mitochondrial	MRPS23	21.8	47	6	2	4	3
Q8WXQ8	Carboxypeptidase A5	CPA5	49	25	3	2	4	3
P24752	Acetyl-CoA acetyltransferase, mitochondrial	ACAT1	45.2	53	6	2	2	1
Q9Y3C1	Nucleolar protein 16	NOP16	21.2	67	9	2	2	3
Q99497	Parkinson disease protein 7	PARK7	19.9	26	6	2	2	2
P06312	Immunoglobulin kappa variable 4-1	IGKV4-1	13.4	83	22	2	7	4
Q9Y487	V-type proton ATPase 116 kDa subunit a2	ATP6V0A2	98	28	2	2	3	2
Q07021	Complement component 1 Q subcomponent-binding protein, mitochondrial	C1QBP	31.3	40	8	2	6	3
Q9Y262	Eukaryotic translation initiation factor 3 subunit L	EIF3L	66.7	33	3	2	6	1
O95470	Sphingosine-1-phosphate lyase 1	SGPL1	63.5	75	5	2	2	2
O75521	Enoyl-CoA delta isomerase 2	ECI2	43.6	122	4	2	6	3
Q15637	Splicing factor 1	SF1	68.3	60	3	2	2	1
P27169	Serum paraoxonase/arylesterase 1	PON1	39.7	35	5	2	7	1
Q13724	Mannosyl-oligosaccharide glucosidase	MOGS	91.9	107	4	2	5	3
P50993	Sodium/potassium-transporting ATPase subunit alpha-2	ATP1A2	112.2	54	2	2	5	3
P27348	14-3-3 protein theta	YWHAQ	27.7	181	20	2	6	4
P18206	Vinculin	VCL	123.7	31	2	2	2	1
Q9BVA0	Katanin p80 WD40 repeat-containing subunit B1	KATNB1	72.3	58	7	2	7	2
Q9BWW9	Apolipoprotein L5	APOL5	47	32	4	2	2	1
Q9H8H0	Nucleolar protein 11	NOL11	81.1	24	2	2	7	2
P68371	Tubulin beta-4B chain	TUBB4B	49.8	773	46	2	2	23
Q9BRP8	Partner of Y14 and mago	PYM1	22.6	40	9	2	4	1
P11908	Ribose-phosphate pyrophosphokinase 2	PRPS2	34.7	101	11	2	2	3
P18669	Phosphoglycerate mutase 1	PGAM1	28.8	49	18	2	6	3
P30101	Protein disulfide-isomerase A3	PDIA3	56.7	40	3	2	6	1
Q9C0J8	pre-mRNA 3' end processing protein WDR33	WDR33	145.8	38	2	2	7	1
Q9Y5G1	Protocadherin gamma-B3	PCDHGB3	101.1	37	3	1	2	2
P18085	ADP-ribosylation factor 4	ARF4	20.5	28	6	1	5	3
Q7KZF4	Staphylococcal nuclease domain-containing protein 1	SND1	101.9	41	3	1	4	2
O00410	Importin-5	IPO5	123.6	70	3	1	4	2
P56270	Myc-associated zinc finger protein	MAZ	48.6	43	3	1	6	1
Q5BKZ1	DBIRD complex subunit ZNF326	ZNF326	65.6	84	5	1	4	2

P23284	Peptidyl-prolyl cis-trans isomerase B	PPIB	23.7	42	8	1	4	1
Q96G21	U3 small nucleolar ribonucleoprotein protein IMP4	IMP4	33.7	90	6	1	3	2
Q13561	Dynactin subunit 2	DCTN2	44.2	35	7	1	1	1
Q6P1J9	Parafibromin	CDC73	60.5	35	5	1	5	3
Q96GM8	Target of EGR1 protein 1	TOE1	56.5	27	3	1	1	2
Q5JTW2	Centrosomal protein of 78 kDa	CEP78	76.3	27	4	1	3	3
P49006	MARCKS-related protein	MARCKSL1	19.5	30	8	1	4	3
P46736	Lys-63-specific deubiquitinase BRCC36	BRCC3	36	38	3	1	3	2
Q8NEJ9	Neuroguidin	NGDN	35.9	49	8	1	4	1
Q15758	Neutral amino acid transporter B(0)	SLC1A5	56.6	52	2	1	2	2
P48444	Coatomer subunit delta	ARCN1	57.2	23	4	1	1	3
Q96DV4	39S ribosomal protein L38, mitochondrial	MRPL38	44.6	40	4	1	2	2
Q9P2J5	Leucine--tRNA ligase, cytoplasmic	LARS1	134.4	46	4	1	1	1
Q8IX18	Probable ATP-dependent RNA helicase DHX40	DHX40	88.5	33	3	1	5	1
Q9Y5B8	Nucleoside diphosphate kinase 7	NME7	42.5	26	4	1	4	2
Q9ULC4	Malignant T-cell-amplified sequence 1	MCTS1	20.5	29	9	1	2	2
P13861	cAMP-dependent protein kinase type II-alpha regulatory subunit	PRKAR2A	45.5	32	5	1	1	1
P16615	Sarcoplasmic/endoplasmic reticulum calcium ATPase 2	ATP2A2	114.7	36	3	1	6	2
Q9UGR2	Zinc finger CCCH domain-containing protein 7B	ZC3H7B	109.8	49	1	1	6	3
P49915	GMP synthase [glutamine-hydrolyzing]	GMPS	76.7	79	4	1	2	2
Q9NZN5	Rho guanine nucleotide exchange factor 12	ARHGEF12	173.1	27	2	1	3	1
Q9Y4W2	Ribosomal biogenesis protein LAS1L	LAS1L	83	26	5	1	4	3
Q9BV38	WD repeat-containing protein 18	WDR18	47.4	31	2	1	1	1
Q9Y512	Sorting and assembly machinery component 50 homolog	SAMM50	51.9	35	3	1	6	3
Q13425	Beta-2-syntrophin	SNTB2	57.9	23	3	1	3	2
O14735	CDP-diacylglycerol--inositol 3-phosphatidyltransferase	CDIPT	23.5	25	6	1	4	3
Q9UHV9	Prefoldin subunit 2	PFDN2	16.6	33	11	1	1	2
Q9H967	WD repeat-containing protein 76	WDR76	69.7	38	1	1	4	2
P51149	Ras-related protein Rab-7a	RAB7A	23.5	26	7	1	6	3
Q9NUQ6	SPATS2-like protein	SPATS2L	61.7	29	4	1	4	1

Table S2 shRNA sequences used in this study

shRNA		5’ to 3’
HMGB1 Sh#1	forward	5'-AGACCUGAGAAUGUAUCCCCAAADdtdt-3'
	reverse	5'-UUUGGGGAUACAUUCUCAGGUCUdTdT-3'

Table S3 PCR primers of USP15 and HMGB1 used in this study

primers		5' to 3'
USP15	forward	5'-ACGCTGCTCAAAACCTCG-3'
	reverse	5'-ACATACCCTGTTCAACCACCT-3'
HMGB1	forward	5'-GCGAAGAAACTGGGAGAGATGTG-3'
	reverse	5'-GCATCAGGCTTTCCTTTAGCTCG--3'
